# Menstrual Hygiene Knowledge, Attitudes, and Practices Among Female Medical Students in Bangladesh: A Cross-Sectional Analysis

**DOI:** 10.7759/cureus.112998

**Published:** 2026-07-20

**Authors:** Israt Jahan Chowdhury, Munmun Hossain, Swarna Paul

**Affiliations:** 1 Department of Physiology, Ad-din Women's Medical College Hospital, Dhaka, BGD; 2 Department of Physiology, Shahabuddin Medical College, Dhaka, BGD; 3 Department of Microbiology, Chittagong Medical College, Chattogram, BGD

**Keywords:** attitudes, knowledge, medical students, menstrual hygiene, reproductive health

## Abstract

Background: Menstrual hygiene management is an important public health issue related to reproductive health, dignity, and well-being. Although medical students are expected to have basic knowledge of reproductive physiology, gaps may persist in practical menstrual hygiene knowledge, attitudes, and practices (KAP). This study aimed to assess KAP regarding menstrual hygiene among second-year female medical students.

Methods: This single-institution cross-sectional study was conducted from January 2025 to June 2025 among second-year female medical students at Shahabuddin Medical College Hospital, Dhaka, Bangladesh. Of 63 eligible female second-year MBBS students, 46 participated and were recruited using convenience sampling. Data were collected using a pre-tested, structured, self-administered questionnaire assessing sociodemographic characteristics and menstrual hygiene-related KAP. Composite KAP scores were calculated. Multiple linear regression analysis was performed to examine factors associated with KAP scores.

Results: The mean age of the participants was 20.98 ± 0.61 years. Twenty-one participants 21 (45.65%) resided in hostels, and 42 (91.30%) were from urban backgrounds. All participants reported awareness of menstruation, and 41 (89.13%) correctly identified the normal menstrual cycle length. Most participants strongly agreed that menstruation is a natural and normal process (42, 91.30%) and that proper menstrual hygiene has health benefits (44, 95.65%). Sanitary pads were the most commonly used menstrual product (43, 93.48%). However, only 26 (56.52%) correctly identified changing menstrual products every two to four hours as appropriate. Hostel residence was significantly associated with higher KAP scores (all p < 0.001). Urban background was also significantly associated with higher knowledge (p < 0.001), attitude (p = 0.005), and practice scores (p < 0.001).

Conclusions: Second-year female medical students demonstrated good general awareness and favorable attitudes regarding menstruation and menstrual hygiene. However, practical gaps remained, particularly regarding appropriate menstrual product-changing frequency and product diversity. Hostel residence and urban background were associated with higher KAP scores, although findings should be interpreted cautiously because of the small sample size and single-institution design. Structured menstrual health education may help improve practical hygiene knowledge and future counseling capacity among medical students.

## Introduction

Menstrual hygiene management is an important component of reproductive and public health among girls and women of reproductive age. Although menstruation is a normal physiological process, misconceptions, stigma, and social restrictions surrounding menstruation continue to affect menstrual hygiene behaviors, access to reliable information, and willingness to discuss menstrual health openly [1,2].

Inadequate menstrual hygiene practices may affect physical comfort, dignity, education, and reproductive health. Menstrual health and hygiene indicators are increasingly recognized as important for public health monitoring, education, and health promotion [3]. These challenges are particularly relevant in low- and middle-income countries, where affordability of menstrual products, access to water, sanitation and hygiene facilities, privacy, disposal systems, and sociocultural beliefs can influence menstrual hygiene practices [2,4,5].

Evidence from Bangladesh suggests that knowledge and practice gaps regarding menstruation and menstrual hygiene persist among reproductive-aged women, even when general awareness is present [5]. Similar findings from college and university settings indicate that formal education alone may not always translate into appropriate menstrual hygiene practices [6,7]. This knowledge-practice gap is important because accurate knowledge, positive attitudes, and appropriate practices are all necessary for effective menstrual hygiene management [8].

Within Bangladesh and similar South Asian settings, menstruation may remain influenced by sociocultural norms that discourage open discussion and contribute to discomfort, misconceptions, or restrictions surrounding menstrual health, even among educated young women [2,5]. Although formal education can improve general knowledge, evidence from university and college populations suggests that theoretical awareness does not always translate into appropriate menstrual hygiene practices or confidence in discussing menstruation [6,8]. These issues are particularly relevant for medical students, who experience menstruation within the same social and cultural environment as their peers while also preparing for professional roles that require open, evidence-based, and culturally sensitive communication about reproductive health.

Female medical students, therefore, represent an important population for menstrual hygiene research. As future physicians and health educators, their knowledge, attitudes, and personal practices may influence not only their own menstrual health but also their confidence and ability to counsel patients, address misconceptions, and communicate about sensitive reproductive health concerns. However, evidence specifically examining menstrual hygiene knowledge, attitudes, and practices (KAP) among female medical students in Bangladesh remains limited. Identifying practical knowledge gaps or persistent discomfort within this comparatively well-educated group may help determine whether routine medical education sufficiently addresses the practical and sociocultural dimensions of menstrual health.

Therefore, this study aimed to assess KAP regarding menstrual hygiene among second-year female medical students at Shahabuddin Medical College Hospital, Dhaka, Bangladesh. The study also explored associations between KAP scores and selected sociodemographic characteristics, including age, hostel residence, urban or rural background, and comorbidity status.

## Materials and methods

Study design and participants

The sampling frame included all 63 female second-year MBBS students enrolled during the study period. Of these, 46 participated, yielding a response proportion of 73.02%. Participants were recruited using convenience sampling based on their availability during data collection. Eligible participants were female second-year MBBS students who provided informed consent. The remaining 17 eligible students were either absent during data collection or declined participation; the specific distribution of reasons for non-participation was not systematically recorded. Because participation was based on availability and willingness to participate, the possibility of selection bias could not be excluded.

Data collection instrument and scoring

Data were collected using an author-developed, structured, self-administered questionnaire. The questionnaire was designed after reviewing relevant menstrual hygiene literature and aligning items with the study objectives. It included sections on sociodemographic characteristics, and KAP regarding menstrual hygiene.

For content validation, the draft questionnaire was reviewed by public health experts for relevance, clarity, and contextual appropriateness. It was pilot tested among 20 female medical students who were not included in the final analysis. Minor wording changes were made after pilot testing to improve clarity. The final questionnaire items and scoring system are shown in Appendix 1-3.

Knowledge was assessed using five objective questions; correct responses were scored as 1 and incorrect or 'do not know' responses as 0, yielding a total score of 0 to 5 (Appendix 1). Attitude was assessed using three Likert-scale items scored from 0 to 4, yielding a total score of 0 to 12 (Appendix 2). Practice was assessed using four items scored from 0 to 2, yielding a total score of 0 to 8 (Appendix 3). Higher scores indicated better knowledge, more positive attitudes, and better menstrual hygiene practices. Medication use during menstruation was recorded descriptively but was not included in the practice score because it reflects symptom management rather than menstrual hygiene behavior.

For descriptive interpretation, KAP scores were converted to percentages and categorized using modified Bloom's cutoff points: good/positive (80-100%), moderate/neutral/fair (60-79%), and poor/negative (<60%). Continuous KAP scores were used in regression analyses.

Ethical considerations

Ethical approval for this study was obtained from the Institutional Ethics Committee of Shahabuddin Medical College Hospital, Dhaka, Bangladesh (Approval No. SMC-A12/25/742; approved on July 24, 2025). Administrative permission was obtained from the college authority prior to data collection. Participation was voluntary, and informed consent was obtained from all participants before enrollment in the study. Participants were assured of confidentiality and anonymity, and no personally identifiable information was collected. All procedures were conducted in accordance with the ethical principles outlined in the Declaration of Helsinki.

Statistical analysis

Data were entered, cleaned, and analyzed using IBM SPSS Statistics for Windows, Version 25 (Released 2017; IBM Corp., Armonk, New York, United States). Continuous variables were summarized using means and standard deviations, whereas categorical variables were presented as frequencies and percentages.

Multiple linear regression analyses were performed to explore factors associated with KAP scores. Four prespecified explanatory variables, age, hostel residence, urban or rural background, and comorbidity status, were included in each model. Given the limited sample size, the regression analyses were considered exploratory, and model complexity was restricted to these four variables. No formal a priori power calculation was performed for the regression analyses; therefore, estimates, particularly those involving small subgroups, were interpreted cautiously. Regression coefficients (B), standard errors (SE), 95% confidence intervals (CI), t statistics, and p-values were reported. Statistical significance of individual regression coefficients was assessed using two-sided t-tests.

Pearson correlation coefficients were calculated to explore associations among demographic variables and KAP scores, and the results were visualized using a correlation heatmap. Because some variables were binary-coded and subgroup sizes were small, correlation results were interpreted descriptively. All statistical tests were two-tailed, and a p-value < 0.05 was considered statistically significant.

## Results

No missing responses were observed for the variables included in the final analysis. Table 1 presents the sociodemographic characteristics of the participants. The mean age of the participants was 20.98 ± 0.61 years. Twenty-one participants (45.65%) resided in hostels, while 25 (54.35%) did not. Most participants were from urban backgrounds 42 (91.30%), and only a small proportion were from rural areas (4, 8.70%). Comorbidity was reported by five (10.87%) participants.

**Table 1:** Sociodemographic characteristics of the participants (n = 46)

Characteristic	Category	n (%) or Mean ± SD
Age, years		20.98 ± 0.61
Hostel residence	Yes	21 (45.65)
	No	25 (54.35)
Residence	Urban	42 (91.30)
	Rural	4 (8.70)
Comorbidity	Yes	5 (10.87)
	No	41 (89.13)

Table 2 summarizes participants' knowledge regarding menstruation and menstrual hygiene. All participants reported awareness of menstruation. Most correctly identified the normal menstrual cycle length (41, 89.13%) and duration of menstrual bleeding (42, 91.30%). The majority considered sanitary pads safe (42, 91.30%), while 26 (56.52%) correctly identified the recommended frequency of changing sanitary pads as every two to four hours.

**Table 2:** Knowledge regarding menstrual hygiene among the participants (n = 46)

Knowledge item	Response	n (%)
Awareness of menstruation	Yes	46 (100.00)
	No	0 (0.00)
Normal duration of the menstrual cycle	21 to 35 days	41 (89.13)
	15 to 20 days	5 (10.87)
	Do not know	0 (0.00)
Normal duration of menstrual bleeding	2 to 7 days	42 (91.30)
	8 to 10 days	4 (8.70)
	Do not know	0 (0.00)
Safety of sanitary pads	Yes	42 (91.30)
	No	1 (2.17)
	Do not know	3 (6.52)
Appropriate frequency of changing sanitary pads	Every 2 to 4 hours	26 (56.52)
	Every 6 to 8 hours	20 (43.48)
	Once daily	0 (0.00)
	Do not know	0 (0.00)

Table 3 presents participants' attitudes toward menstruation and menstrual hygiene. Nearly all participants strongly agreed that menstruation is a natural and normal process (42, 91.30%). Most participants also strongly agreed that proper menstrual hygiene has health benefits (44, 95.65%). However, attitudes toward discussing menstruation were more varied, with approximately one-third reporting neutrality or discomfort.

**Table 3:** Attitudes toward menstrual hygiene among the participants (n = 46)

Attitude item	Response	n (%)
Menstruation is a natural and normal process	Strongly agree	42 (91.30)
	Agree	4 (8.70)
	Neutral	0 (0.00)
	Disagree	0 (0.00)
	Strongly disagree	0 (0.00)
Comfortable discussing menstruation	Strongly agree	21 (45.65)
	Agree	11 (23.91)
	Neutral	9 (19.57)
	Disagree	4 (8.70)
	Strongly disagree	1 (2.17)
Proper menstrual hygiene has health benefits	Strongly agree	44 (95.65)
	Agree	2 (4.35)
	Neutral	0 (0.00)
	Disagree	0 (0.00)
	Strongly disagree	0 (0.00)

Table 4 summarizes menstrual hygiene practices among the participants. Sanitary pads were the predominant menstrual product used (43, 93.48), while three (6.52%) reported using menstrual cups. More than half of the participants changed menstrual products every two to four hours. All participants reported handwashing before and after changing menstrual products and disposing of used products in dustbins. Most participants did not use medication for menstruation-related symptoms.

**Table 4:** Menstrual hygiene practices among the participants (n = 46) PCOS: polycystic ovary syndrome

Practice item	Response	n (%)
Menstrual product used	Sanitary pads	43 (93.48)
	Cloth	0 (0.00)
	Menstrual cup	3 (6.52)
	Tampon	0 (0.00)
Frequency of changing menstrual products	Every 2 to 4 hours	26 (56.52)
	Every 6 to 8 hours	20 (43.48)
	Once daily	0 (0.00)
Handwashing before and after changing menstrual products	Always	46 (100.00)
	Sometimes	0 (0.00)
	Never	0 (0.00)
Disposal of used menstrual products	Dustbin	46 (100.00)
	Toilet	0 (0.00)
	Burning	0 (0.00)
Medication use for menstruation-related pain or discomfort	No medication	30 (65.22)
	Regular medication	4 (8.70)
	Irregular medication	10 (21.74)
	Doctor-prescribed regular medication for PCOS	2 (4.35)

Table 5 presents the multiple linear regression analysis examining factors associated with knowledge scores regarding menstrual hygiene. Hostel residence and urban background were significantly associated with higher knowledge scores. Age and comorbidity were not significantly associated with knowledge scores.

**Table 5:** Multiple linear regression analysis for knowledge score regarding menstrual hygiene B: unstandardized regression coefficient; SE: standard error; CI: confidence interval

Variable	B	SE	t-value	p-value	95% CI
Intercept	11.82	8.38	1.41	0.166	-5.11 to 28.75
Age	-0.47	0.40	-1.17	0.248	-1.28 to 0.34
Hostel residence, yes vs no	1.19	0.19	6.43	<0.001	0.82 to 1.57
Urban residence, urban vs rural	1.83	0.31	6.00	<0.001	1.21 to 2.45
Comorbidity, yes vs no	-0.03	0.29	-0.09	0.929	-0.61 to 0.55

Table 6 shows the multiple linear regression analysis for attitude scores regarding menstrual hygiene. Both hostel residence and urban background demonstrated significant positive associations with attitude scores. No significant associations were observed for age or comorbidity.

**Table 6:** Multiple linear regression analysis for attitude score regarding menstrual hygiene B: unstandardized regression coefficient; SE: standard error; CI: confidence interval

Variable	B	SE	t-value	p-value	95% CI
Intercept	9.46	5.47	1.73	0.091	-1.58 to 20.51
Age	-0.16	0.26	-0.61	0.546	-0.69 to 0.37
Hostel residence, yes vs no	0.53	0.13	4.08	<0.001	0.26 to 0.80
Urban residence, urban vs rural	0.62	0.21	2.95	0.005	0.20 to 1.05
Comorbidity, yes vs no	-0.05	0.19	-0.26	0.795	-0.44 to 0.34

Table 7 presents the multiple linear regression analysis for practice scores regarding menstrual hygiene. Similar to the findings for knowledge and attitude scores, hostel residence and urban background were significantly associated with higher practice scores. Age and comorbidity were not significantly associated with practice scores.

**Table 7:** Multiple linear regression analysis for practice score regarding menstrual hygiene B: unstandardized regression coefficient; SE: standard error; CI: confidence interval

Variable	B	SE	t-value	p-value	95% CI
Intercept	14.23	6.32	2.25	0.030	1.49 to 26.97
Age	-0.43	0.30	-1.43	0.160	-1.04 to 0.18
Hostel residence, yes vs no	0.85	0.16	5.31	<0.001	0.53 to 1.18
Urban residence, urban vs rural	1.02	0.26	3.92	<0.001	0.50 to 1.54
Comorbidity, yes vs no	-0.10	0.24	-0.41	0.684	-0.59 to 0.39

**Figure 1:**
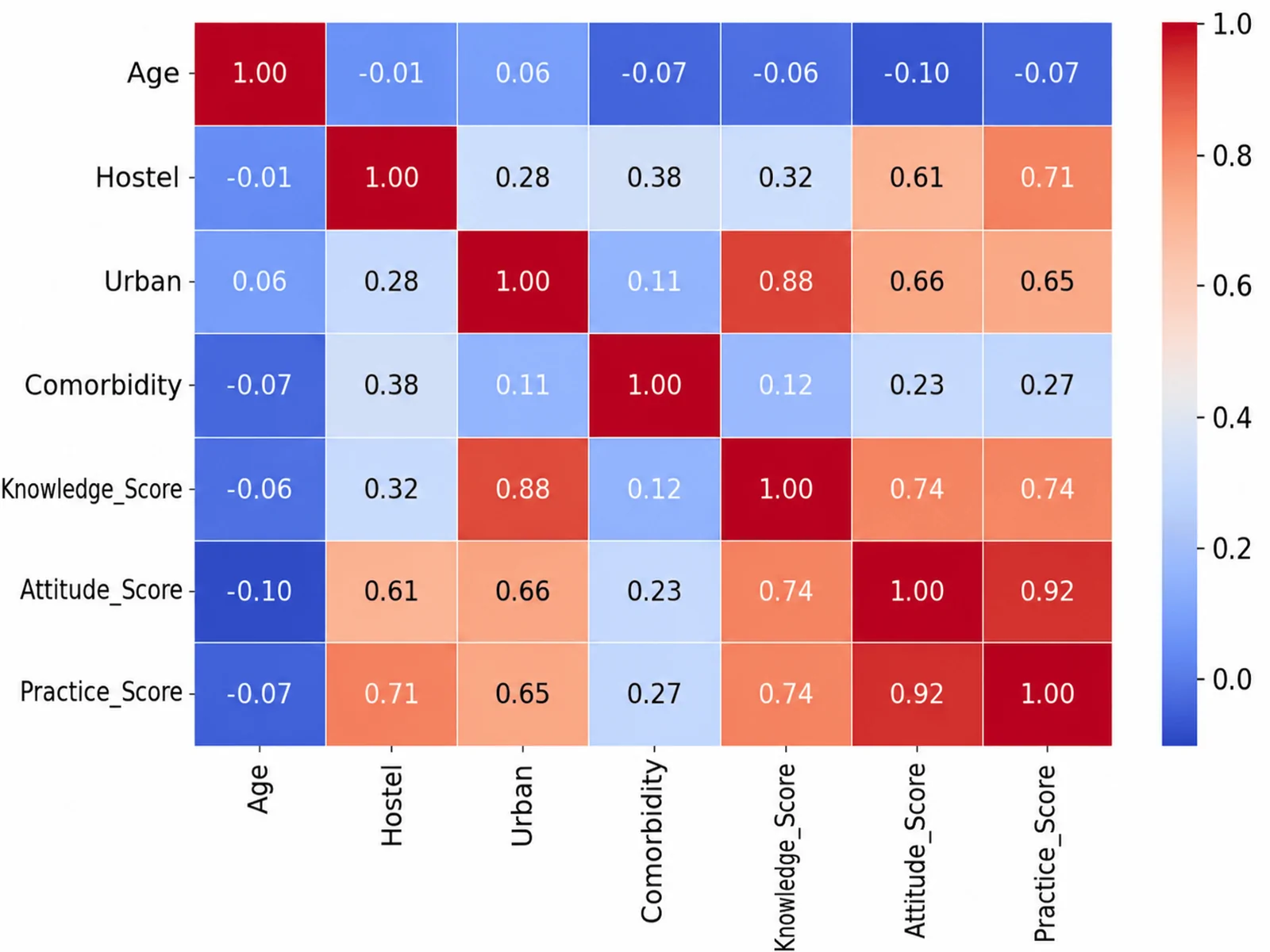
Correlation heatmap of demographic variables and KAP scores KAP: knowledge, attitudes, or practices

Correlation analysis showed that KAP scores were positively correlated with each other. The strongest correlation was observed between attitude and practice scores (r = 0.92). Knowledge score was positively correlated with attitude score (r = 0.74) and practice score (r = 0.74). Urban background showed a strong positive correlation with knowledge score (r = 0.88) and moderate positive correlations with attitude and practice scores (r = 0.66 and r = 0.65, respectively). Hostel residence was positively correlated with attitude and practice scores (r = 0.61 and r = 0.71, respectively). Age and comorbidity showed weak correlations with KAP scores. These correlations are descriptive and should be interpreted cautiously because of the small sample size and unbalanced subgroup distribution (Figure 1).

## Discussion

This cross-sectional study assessed KAP regarding menstrual hygiene among second-year female medical students. Overall, participants demonstrated good general awareness of menstruation and favorable attitudes toward menstrual hygiene. However, gaps remained in practical knowledge and behavior, particularly regarding the recommended frequency of changing menstrual products and the limited diversity of menstrual product use. These findings suggest that general awareness of menstruation does not necessarily ensure comprehensive menstrual hygiene practice.

All participants were aware of menstruation, and most correctly identified the normal duration of the menstrual cycle and menstrual bleeding. This level of knowledge may reflect the educational background of the participants, as medical students are expected to have some exposure to reproductive physiology. However, the finding that 20 (43.48%) of participants selected changing sanitary pads every six to eight hours indicates a practical knowledge gap. This is consistent with previous research from Bangladesh, which reported that knowledge and practice gaps regarding menstrual hygiene may persist even among reproductive-aged women with general awareness of menstruation [5]. Similar gaps have also been reported in student populations, indicating that menstrual hygiene education should emphasize practical behavior in addition to theoretical knowledge [6,7].

Participants' attitudes toward menstruation were generally positive. Most strongly agreed that menstruation is a natural and normal process, and nearly all recognized the health benefits of proper menstrual hygiene. However, comfort with discussing menstruation was more varied, with a notable proportion of students reporting neutral or uncomfortable responses. This finding suggests that social discomfort surrounding menstruation may persist even among medical students. Such discomfort is important because future physicians need to communicate openly and sensitively about menstrual and reproductive health. Reducing stigma and improving communication skills should therefore be considered part of menstrual health education in medical training [2,7].

The reported hygiene practices showed both strengths and areas for improvement. Most participants used sanitary pads, and all reported handwashing before and after changing menstrual products and disposal of used products in dustbins. These findings indicate favorable self-reported hygiene behaviors. However, the predominance of sanitary pad use, with only a small proportion reporting menstrual cup use and no reported use of tampons or cloth, suggests limited diversity in menstrual product use. This may reflect personal preference, cultural acceptability, product availability, affordability, or limited exposure to alternative menstrual products. Menstrual health education should therefore include balanced, evidence-based information on available menstrual products, safe use, hygiene requirements, and disposal considerations.

Hostel residence and urban background were significantly associated with higher KAP scores. Students living in hostels may have greater opportunities for peer discussion, shared experiences, and access to institutional hygiene facilities. Similarly, students from urban backgrounds may have greater exposure to health information, menstrual products, and healthcare resources. These findings are consistent with previous studies suggesting that environmental conditions, access to facilities, and social context can influence menstrual hygiene practices [6,9,10]. However, these associations should be interpreted cautiously because the study included a small sample and only four participants from rural backgrounds.

Age and comorbidity were not significantly associated with KAP scores. This may be partly explained by the narrow age range and relatively homogeneous academic background of the participants. The small number of participants with comorbidities also limits meaningful interpretation of this variable. Therefore, the absence of statistically significant associations should not be interpreted as evidence that these factors have no role in menstrual health behavior.

Medication use for menstruation-related pain or discomfort was recorded descriptively and was not included in the practice score. Most participants reported no medication use, while others reported regular, irregular, or doctor-prescribed medication use. This finding should be interpreted cautiously because the study did not assess pain severity, dysmenorrhea diagnosis, indications for medication, or access to medical consultation. Nevertheless, it highlights the relevance of including menstrual symptom recognition and evidence-based management of menstrual discomfort within broader menstrual health education [11,12].

The findings have implications for medical education. Although the participants showed favorable general knowledge and attitudes, practical gaps remained. Medical curricula and institutional health programs should include structured menstrual health education covering menstrual physiology, hygiene practices, menstrual product options, disposal practices, symptom management, and communication about menstruation. Peer education, small-group discussions, and practical demonstrations may also help reduce discomfort and improve confidence in discussing menstrual health. Institutional support, including access to appropriate hygiene facilities and menstrual products, may further strengthen menstrual hygiene practices among students.

Limitations of the study

This study has several limitations. First, the sample was small and was drawn from a single medical college; therefore, the findings may not be generalizable to all female medical students in Bangladesh or other settings. Convenience sampling and a response proportion of 73.02% may also have introduced selection bias. Because the specific distribution of reasons for non-participation was not systematically recorded, differences between participants and non-participants could not be assessed.

Second, the regression analyses were exploratory, and no formal a priori power calculation was performed for the regression models. The small overall sample and highly unbalanced subgroups, particularly the four participants from rural backgrounds and five participants with comorbidities, may have reduced statistical power and the stability of coefficient estimates. Accordingly, the observed associations should be interpreted cautiously and require confirmation in larger studies.

Third, the cross-sectional design prevents causal interpretation of the observed associations. Data were collected using a self-administered questionnaire and may therefore have been affected by recall and social desirability bias, particularly for sensitive hygiene-related behaviors. Although the questionnaire was developed through literature review, expert review, and pilot testing, it was not psychometrically validated in this population. In addition, the absence of variability in some practice items, particularly handwashing and disposal practices, limited their ability to differentiate participants. Several potentially important factors were also not assessed, including socioeconomic status, parental education, sources of menstrual information, previous menstrual health education, access to water and sanitation facilities, affordability of menstrual products, and severity of menstrual symptoms.

## Conclusions

This study found that second-year female medical students had good general awareness and favorable attitudes regarding menstruation and menstrual hygiene. However, practical gaps were identified, particularly in knowledge and practice related to the frequency of changing menstrual products and limited exposure to alternative menstrual products. Hostel residence and urban background were associated with higher KAP scores, although these findings should be interpreted cautiously because of the small sample size and single-institution design.

The findings support the need for structured menstrual health education in medical training, with emphasis on practical hygiene behaviors, menstrual product awareness, disposal practices, symptom management, and open communication about menstrual health. Future studies should include larger, multi-institutional samples and qualitative methods to explore social, cultural, and institutional barriers to appropriate menstrual hygiene among medical students.

## Appendices

The following questionnaire items were used to generate the KAP scores. Responses were self-reported. Medication use during menstruation was recorded descriptively and was not included in the practice score.

Appendix 1

**Table 8:** Knowledge items, response options, and scoring

Item	Response options	Scoring
Awareness of menstruation	Yes	1
	No	0
Normal duration of menstrual cycle	21–35 days	1
	15–20 days	0
	Do not know	0
Normal duration of menstrual bleeding	2–7 days	1
	8–10 days	0
	Do not know	0
Safety of sanitary pads	Yes	1
	No	0
	Do not know	0
Appropriate frequency of changing sanitary pads	Every 2–4 hours	1
	Every 6–8 hours	0
	Once daily	0
	Do not know	0

Appendix 2

**Table 9:** Attitude items, response options, and scoring

Item	Response option	Scoring
Menstruation is a natural and normal process	Strongly disagree	0
	Disagree	1
	Neutral	2
	Agree	3
	Strongly agree	4
Comfortable discussing menstruation	Strongly disagree	0
	Disagree	1
	Neutral	2
	Agree	3
	Strongly agree	4
Proper menstrual hygiene has health benefits	Strongly disagree	0
	Disagree	1
	Neutral	2
	Agree	3
	Strongly agree	4

Appendix 3

**Table 10:** Practice items, response options, and scoring

Item	Response option	Scoring
Menstrual product used	Sanitary pads	2
	Menstrual cup	2
	Tampon	2
	Clean reusable cloth	1
	Unsafe material	0
Frequency of changing menstrual products	Every 2–4 hours	2
	Every 6–8 hours	1
	Once daily	0
Handwashing before and after changing menstrual products	Always	2
	Sometimes	1
	Never	0
Disposal of used menstrual products	Dustbin	2
	Burning	1
	Toilet	0
	Open environment	0
